# Sulfur cycling likely obscures dynamic biologically‐driven iron redox cycling in contemporary methane seep environments

**DOI:** 10.1111/1758-2229.13263

**Published:** 2024-05-05

**Authors:** Isabel R. Baker, Peter R. Girguis

**Affiliations:** ^1^ Department of Organismic and Evolutionary Biology Harvard University Cambridge Massachusetts USA; ^2^ Department of Earth and Planetary Science Johns Hopkins University Baltimore Maryland USA

## Abstract

Deep‐sea methane seeps are amongst the most biologically productive environments on Earth and are often characterised by stable, low oxygen concentrations and microbial communities that couple the anaerobic oxidation of methane to sulfate reduction or iron reduction in the underlying sediment. At these sites, ferrous iron (Fe^2+^) can be produced by organoclastic iron reduction, methanotrophic‐coupled iron reduction, or through the abiotic reduction by sulfide produced by the abundant sulfate‐reducing bacteria at these sites. The prevalence of Fe^2+^in the anoxic sediments, as well as the availability of oxygen in the overlying water, suggests that seeps could also harbour communities of iron‐oxidising microbes. However, it is unclear to what extent Fe^2+^ remains bioavailable and in solution given that the abiotic reaction between sulfide and ferrous iron is often assumed to scavenge all ferrous iron as insoluble iron sulfides and pyrite. Accordingly, we searched the sea floor at methane seeps along the Cascadia Margin for microaerobic, neutrophilic iron‐oxidising bacteria, operating under the reasoning that if iron‐oxidising bacteria could be isolated from these environments, it could indicate that porewater Fe^2+^ can persist is long enough for biology to outcompete pyritisation. We found that the presence of sulfate in our enrichment media muted any obvious microbially‐driven iron oxidation with most iron being precipitated as iron sulfides. Transfer of enrichment cultures to sulfate‐depleted media led to dynamic iron redox cycling relative to abiotic controls and sulfate‐containing cultures, and demonstrated the capacity for biogenic iron (oxyhydr)oxides from a methane seep‐derived community. 16S rRNA analyses revealed that removing sulfate drastically reduced the diversity of enrichment cultures and caused a general shift from a Gammaproteobacteria‐domainated ecosystem to one dominated by *Rhodobacteraceae* (*Alphaproteobacteria*). Our data suggest that, in most cases, sulfur cycling may restrict the biological “ferrous wheel” in contemporary environments through a combination of the sulfur‐adapted sediment‐dwelling ecosystems and the abiotic reactions they influence.

## Graphical abstract

Marine methane seeps are highly productive seafloor sites driven in part by a robust sulfur cycle. The impact that sulfur has on the cycling of bioavailable iron is poorly understood. One indicator of iron's bioavailability would be the persistence of iron‐oxidising bacteria, as they must rely on bioavailable Fe(II) for energy. As such, we attempted to enrich iron‐oxidising microbes from a deep‐sea methane seep and found that biologically‐driven iron cycling could only be detected in sulfate‐depleted media, suggesting that iron‐oxidising bacteria are unable to compete with sulfur cycling in a quantitatively significant manner in methane seep sediments.

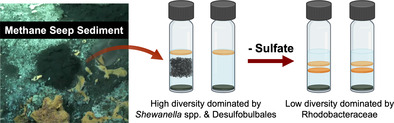

## INTRODUCTION

Marine sediments function as a dynamic reservoir of organic carbon for our planet's oceans (Atwood et al., 2020; Estes et al., 2019; Mcleod et al., 2011). Methane (CH_4_) seepage from these sediments is a significant source of carbon in the marine environment, although the amount released could be catastrophic for our planet's climate if not curtailed (Reeburgh, 2007). The marine sulfur cycle plays a critical role in controlling the flux of CH_4_ out of the seafloor. At the surface of the seafloor and in the sediment just below, aerobic heterotrophs and sulfur‐oxidising bacteria (SOB) quickly deplete oxygen (O_2_) (Glud, 2008; Jørgensen et al., 2019; Jørgensen & Revsbech, 1983). Sulfate‐rich porewater dominates the upper layers of the anoxic sediment, maintained by diffusion of the overlying sulfate‐replete seawater and sulfur re‐oxidation by SOB (Jørgensen et al., 2019 and refs. therein). As sulfate (SO_4_^2−^) diffuses down through the sediment, it overlaps with an upward‐diffusing flux of methane. The horizon where these two chemical gradients meet—the so‐called sulfate–methane transition zone (SMTZ)—is characterised by the local minima of SO_4_^2−^ and CH_4_ concentrations, with SO_4_^2−^ decreasing and CH_4_ increasing with sediment depth (e.g., Niewöhner et al., 1998). This is thanks to the syntrophic coupling between the anaerobic methane‐oxidising archaea (ANME) and the sulfate‐reducing bacteria (SRB) (Hoehler et al., 1994; Hoehler & Alperin, 1996; Joye et al., 2004; Knittel & Boetius, 2009; Reeburgh, 2007). Their syntrophy results in the 1:1 stoichiometric anaerobic oxidation of CH_4_ (AOM) to carbon dioxide (CO_2_) and the reduction of SO_4_^2−^ to sulfide (HS^−^) conventionally represented as the net reaction in equation 1.

(1) $${\mathrm{CH}}_{4}+{{\mathrm{SO}}_{4}}^{2-}\to {{\mathrm{HCO}}_{3}}^{-}+{\mathrm{HS}}^{-}+H_{2}O$$

However, these biological reactions take place amongst a backdrop of other elemental cycles that can influence the availability of reactants and the rate of their interactions. Iron is particularly interesting due to the high reactivity between some Fe and S species (Riedinger et al., 2017). Sulfate can abiotically reduce ferric iron like iron (oxyhydr)oxides (Fe(OH)_3_) to Fe^2+^ (Equation 2) (Hansel et al., 2015; Li et al., 2006; Yao & Millero, 1996), and Fe^2+^ can further react with HS^−^ to form solid FeS (Equation 3), which is usually buried as pyrite (FeS_2_) through further interactions with reduced sulfur species.

(2) $${\mathrm{HS}}^{-}+\text{2Fe}{\left(\mathrm{OH}\right)}_{3}+{\text{5H}}^{+}\to {\text{2Fe}}^{2+}+S_{0}+{\text{6H}}_{2}O$$

(3) $${\mathrm{Fe}}^{2+}+{\mathrm{HS}}^{-}\to \mathrm{FeS}+H^{+}$$

Ferrous iron can also be generated biologically as well. Heterotrophic iron‐reducing bacteria (FeRB) that produce Fe^2+^ using electrons from oxidising organic matter or hydrogen are also well‐documented residents of marine sediment, typically found above the SMTZ but below the reach of O_2_ (Hansel et al., 2015; Otte et al., 2018). Likewise, oxidised iron can serve as an alternative electron acceptor for AOM (FeR‐AOM, equation 4) (Aromokeye et al., 2020; Beal et al., 2009; Cai et al., 2018), although it is unclear how pervasive this may be in contemporary marine environments (Riedinger et al., 2014; Wankel et al., 2012). The unbounded influence and magnitude of FeR‐AOM—and thus its impact on CH_4_ flux—in modern marine seeps can be attributed to two major uncertainties: (A) our inability to differentiate abiogenic Fe^2+^ and biogenic Fe^2+^ and (B) our lack of knowledge on what fraction of Fe^2+^ gets re‐oxidised instead of being scavenged as FeS.

(4) $${\mathrm{CH}}_{4}+\text{8Fe}{\left(\mathrm{OH}\right)}_{3}+15H^{+}\to {{\mathrm{HCO}}_{3}}^{-}+{\text{8Fe}}^{2+}+21H_{2}O$$

Put simply, we do not know the degree to which the Fe cycle can compete with the S cycle in methane seep environments. In this study, we sought to address this gap in our knowledge by assessing the capacity for biological iron‐redox cycling in community enrichments from a marine methane seep. We directed our attention towards iron‐oxidising bacteria (FeOB) and their impact on iron mineralisation, with the assumption that if FeOB are able to persist and impact Fe‐redox cycling in a sulfur‐adapted ecosystem (i.e., compete with Fe^2+^ scavenging by HS^−^), it could serve as a preliminary indication that the Fe^2+^ at methane seeps can persist for further redox‐cycling that would support heterotrophic FeRB or FeR‐AOM, particularly at depths where these metabolic processes might not have been thought to occur. Marine iron‐oxidising bacteria that couple Fe^2+^ oxidation to the reduction of O_2_ (microaerobic FeOB) or nitrate (anaerobic FeOB) have been isolated from a number of seafloor environments (Barco et al., 2017; Blackwell et al., 2020; Edwards et al., 2003, 2004; Emerson et al., 2007; Fleming et al., 2013; Laufer, Niemeyer, et al., 2017; Laufer, Nordhoff, et al., 2017; Makita et al., 2017; Mori et al., 2017; Straub et al., 1996; Widdel et al., 1993), but to our knowledge, have not yet been isolated from a methane seep. Here, we describe our efforts to enrich and characterise FeOB from a methane seep—and their impacts on iron mineralisation—in the presence and absence of sulfate.

## EXPERIMENTAL PROCEDURES

### Sampling and site description

The original inocula for our enrichment cultures came from microbial mat and loose sediment collected with suction sampling by *ROV SuBastian* in August 2018 (Schmidt Ocean Institute Hunting Bubbles Cruise #FK180824). Samples were collected from actively bubbling sites on the seafloor at the McArthur Promontory methane seep in the Cascadia Margin (45.848° N, 124.894° W) at a depth of ~834 metres. Data collected during a CTD rosette cast (Seabird SBE9 with 10 L Niskin water sampling bottles) at approximately the same geographic coordinates indicated dysoxic conditions of 7.95 μmol•kg^−1^ dissolved O_2_ at the seafloor‐water interface. The low O_2_ concentration observed here is consistent with the high rates of biological activity typical of methane seeps (Boetius & Wenzhöfer, 2013; Glud, 2008; Levin et al., 2016) and would also satisfy the environmental niche constraint favoured by microaerobic iron‐oxidising bacteria, should they be present (Maisch et al., 2019; Otte et al., 2018).

While we expected the surface of the seafloor to be relatively firm, we found that the sites we chose to sample were loose and porous. Suction sampling of the white, brown and orange‐yellow microbial mat and surface sediment revealed a soft, gelatinous black material that continued for at least 10 cm before the suction sampler hit anything more solid (Figure S1). Due to the consistency of the seafloor material, discrete sampling proved challenging, and as such, the source material for all our enrichment cultures comprises a mixture of the debris and microbial mats on the seafloor and the black material that lay immediately beneath them. Upon recovering samples, we found that the black material was indeed extremely viscous and mucoid in nature compared to the otherwise powdery and easy‐to‐dissociate seafloor‐surface material that accompanied it.

### Media and culturing conditions

The base media used in all experiments was made according to the protocol for preparing artificial seawater for iron‐oxidising bacteria (FeOB ASW) from the National Center for Marine Algae and Microbiota (NCMA Medium 1). FeOB ASW is constituted of, per litre of distilled water, 27.5 g NaCl, 5.38 g MgCl_2_, 6.78 g MgSO_4_∙7H_2_O, 0.72 g KCl, 1 g NH_4_Cl, 1.4 g CaCl_2_∙2H_2_O, 0.05 g K_2_HPO_4_ and 0.84 g NaHCO_3_, plus final concentrations of 0.1% MD‐TMS Trace Mineral Supplement (ATCC) and 0.1% MD‐VS Vitamin Supplement (ATCC). In sulfate‐depleted experiments, MgSO_4_∙7H_2_O was withheld from the media, though it should be noted that 15.7 μM of sulfate was present in all experiments due to the sulfate salts present in the trace mineral solution. This concentration of sulfate is still four orders of magnitude lower than concentration of the sulfate‐containing media, which contains an additional 27 mM sulfate. The pH of the media was adjusted to pH 6.5 by bubbling with filtered CO_2_:N_2_ (20:80).

All cultures were grown in either semi‐solid media in gradient tubes with an ambient air headspace or in liquid media in petri dishes under an atmosphere of filtered O_2_:CO_2_:N_2_ (8:10:82). Sterile petri dishes were filled with 20 mL FeOB ASW and 0.2 g gamma‐irradiated zero‐valent iron (ZVI), then inoculated with 200 μL gradient tube material. Gradient tubes were prepared as previously described (Emerson & Moyer, 1997; Lueder et al., 2018). Briefly, gamma‐irradiated zero‐valent iron powder (−200 mesh, Thermo Fisher Scientific, irradiated at an average rate of 171 rad/min over 3 days) was added to sterile 7.39 mL borosilicate glass vials. 4 mL of warm FeOB ASW containing 0.15% low‐melt agarose was carefully added overtop the iron powder; tubes were then sealed with rubber stoppers and allowed to set for 4 h at ambient temperature before inoculation. The original inocula (environmental samples) were processed immediately after recovery aboard the *R/V Falkor*. After vortexing, aliquots of each sample were injected into gradient tubes to inoculate the first generation of enrichment cultures. At the end of the cruise, the top 3.9 mL was carefully removed from each gradient tube, so as to not disturb the remaining 100 μL directly overlaying the ZVI. Gradient tube aliquots were then transported back to the lab at 4°C. Upon arrival, these aliquots were immediately homogenised by vortexing, and sub‐samples were injected into freshly prepared gradient tubes.

### 16S sequencing

DNA for 16S amplicon sequencing was extracted from 1‐month‐old gradient tubes. Under sterile conditions, the entirety of the 4 mL gradient tube volume was vortexed, then centrifuged at 2500•g for 10 min. The pellet was then washed with 1 mL sterile 50 mM phosphate buffered saline to adsorb remaining free Fe^2+^ that could otherwise interfere with downstream sequencing. The washed suspension was centrifuged again for 5 min, the supernatant discarded and the resulting pellet weighed before proceeding with DNA extraction with the DNeasy® PowerSoil® Kit according to manufacturer instructions (QIAGEN). Extracted DNA was then sent to the Dalhousie University Integrated Microbiome Resource for PCR amplification and MiSeq (Illumnia) sequencing of the 16S rRNA V4‐V5 hypervariable region.

Raw sequencing reads were processed in R using the DADA2 pipeline version 1.12. DADA2 was also used to assign taxonomy to amplicon sequence variants (ASVs) using version 132 of the Silva taxonomic training set. Those that remained unclassified were searched against the NCBI nucleotide collection (as of January 30, 2022) using BLAST. ASVs were then filtered and analysed using the R package *phyloseq* version 1.28. ASVs were filtered to only include those that were present in a minimum of 2% of samples, members of phyla that appeared in at least two samples, and had at least 20 reads.

## RESULTS

### Enrichment culturing

Gradient tubes are commonly used to enrich for and isolate neutrophilic iron‐oxidising bacteria (see Experimental Procedures for culturing details) (Emerson & Moyer, 1997). Typically, an iron source (in this case, zero‐valent iron powder) at the bottom of a culture tube allows ferrous iron to diffuse upward. Oxygen diffuses down from the headspace, creating a gradient opposing that of Fe^2+^. Both chemical gradients are stabilised by a semi‐solid artificial seawater media. Should iron‐oxidising bacteria be present in the inoculum, they will form a band at their preferred interface between these opposing chemical gradients (Emerson & Moyer, 1997; Lueder et al., 2018). Successive dilution‐to‐extinction cultures are typically required to enrich for and eventually isolate iron‐oxidisers. The enrichment cultures described throughout this paper are called 13A, 13B, and 15A. 13A and 13B were originally inoculated with different aliquots from the same suction sample, while 15A was inoculated with material from a different sample collected at the same site.

**FIGURE 1:**
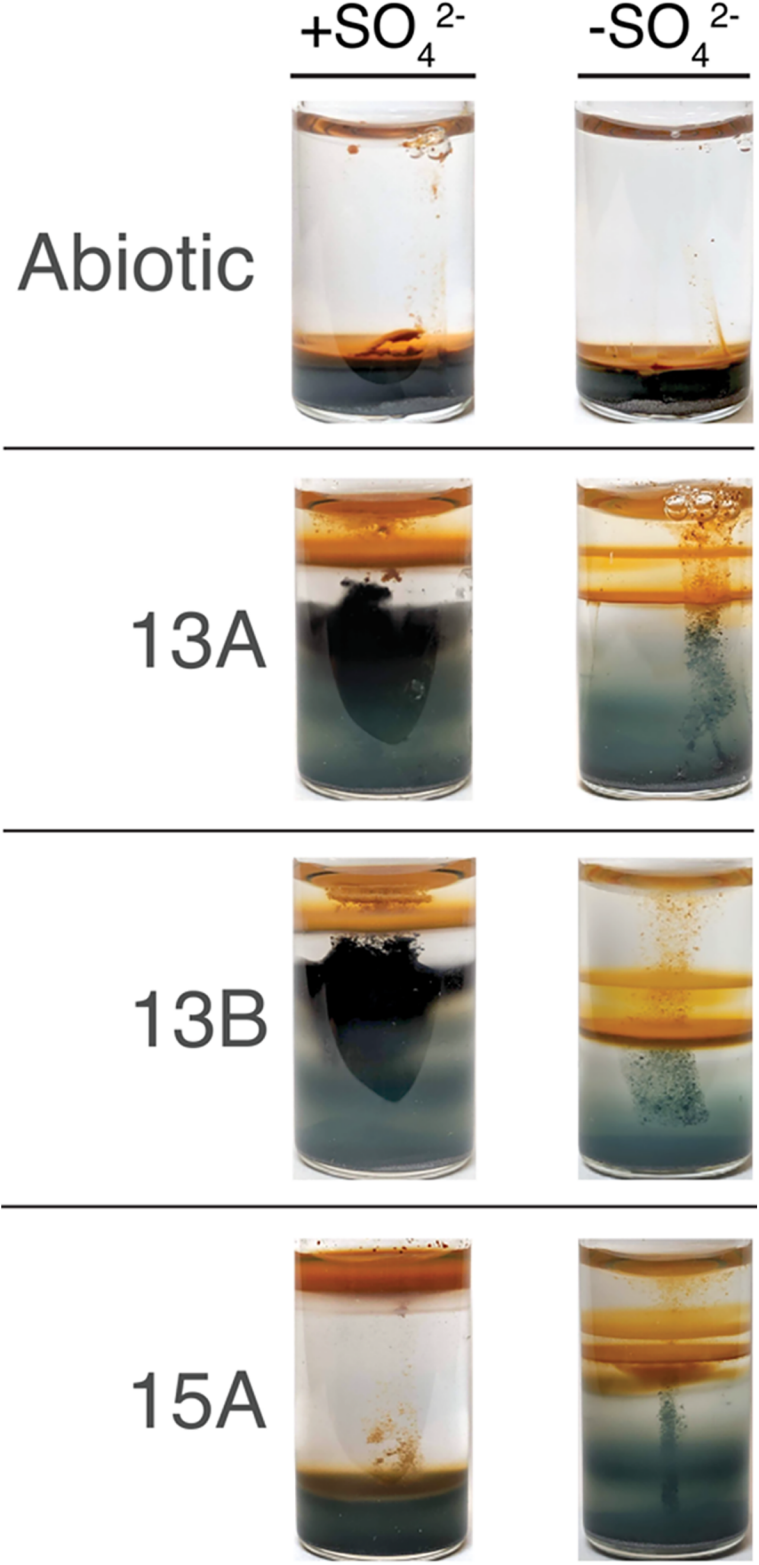
Gradient tube incubations with (left) and without (right) sulfate. Images were taken after incubating for 1‐month in the dark at 20°C. The tubes shown here are the fifth generation of their respective enrichments in either media.

We inoculated each generation of gradient tubes for 1 month before collecting material to inoculate the next generation of enrichment cultures. Over each successive generation, the 13A and 13B gradient tubes consistently developed an orange‐red band (likely iron (oxyhydr)oxides) near the media‐headspace interface and a large horizon of black material below (Figure 1, left column). The black material was never observed above the orange band in these cases and there was always a clear gap with no obvious mineralisation between the two types of precipitates. Like the 13A and 13B lineages, 15A also consistently maintained a rusty horizon near the surface of the media, but unlike the other enrichments, the space below remained clear over the entire course of each month‐long incubation (Figure 1, left column).

EDS analysis later revealed that the black material in 13A and 13B was an iron–sulfur mineral (Figure S2), most likely iron(II) sulfide (FeS). Given that sulfate‐reducing microorganisms (SRM) are abundant at methane seeps and that black precipitates are often used an indicator for SRM (Blackwell et al., 2020; Picard et al., 2016, 2019; van Dongen et al., 2007), we suspected that these minerals were the product of Fe^2+^ in the system reacting with sulfide produced by SRM. A recent study describing a novel gradient tube enrichment technique for co‐culturing SRM and FeOB observed a similar phenomenon, where black precipitates were only formed in the presence of SRM (Brooks & Field, 2021). We thus started a new lineage of cultures in sulfate‐depleted media (see Methods for details) to assess if the black material was indeed generated by the activity of SRM. Cultures in sulfate‐depleted media are hereon labelled with ‘sd’ (e.g., 13A^sd^) to differentiate them from their sulfate‐replete counterparts.

### Establishing lineages under sulfate‐depleted conditions

We observed that the black material gradually disappeared from successive generations of 13A^sd^ and 13B^sd^ (Figure 1, right column) and multiple new, co‐occurring iron‐oxidising fronts formed at different depths, all of which were deeper than the single iron‐oxidising horizon in their sulfate‐containing predecessors. Although the sulfate‐containing cultures of 15A never formed the FeS minerals produced in 13A and 13B, transitioning 15A to sulfate‐depleted media also resulted in a deepening and multiplication of iron‐oxidising fronts. This suggests that even in the absence of obvious mineral precipitation, the presence of sulfate was in some way restraining the iron redox cycling that occurred in the absence of sulfate.

**FIGURE 2:**
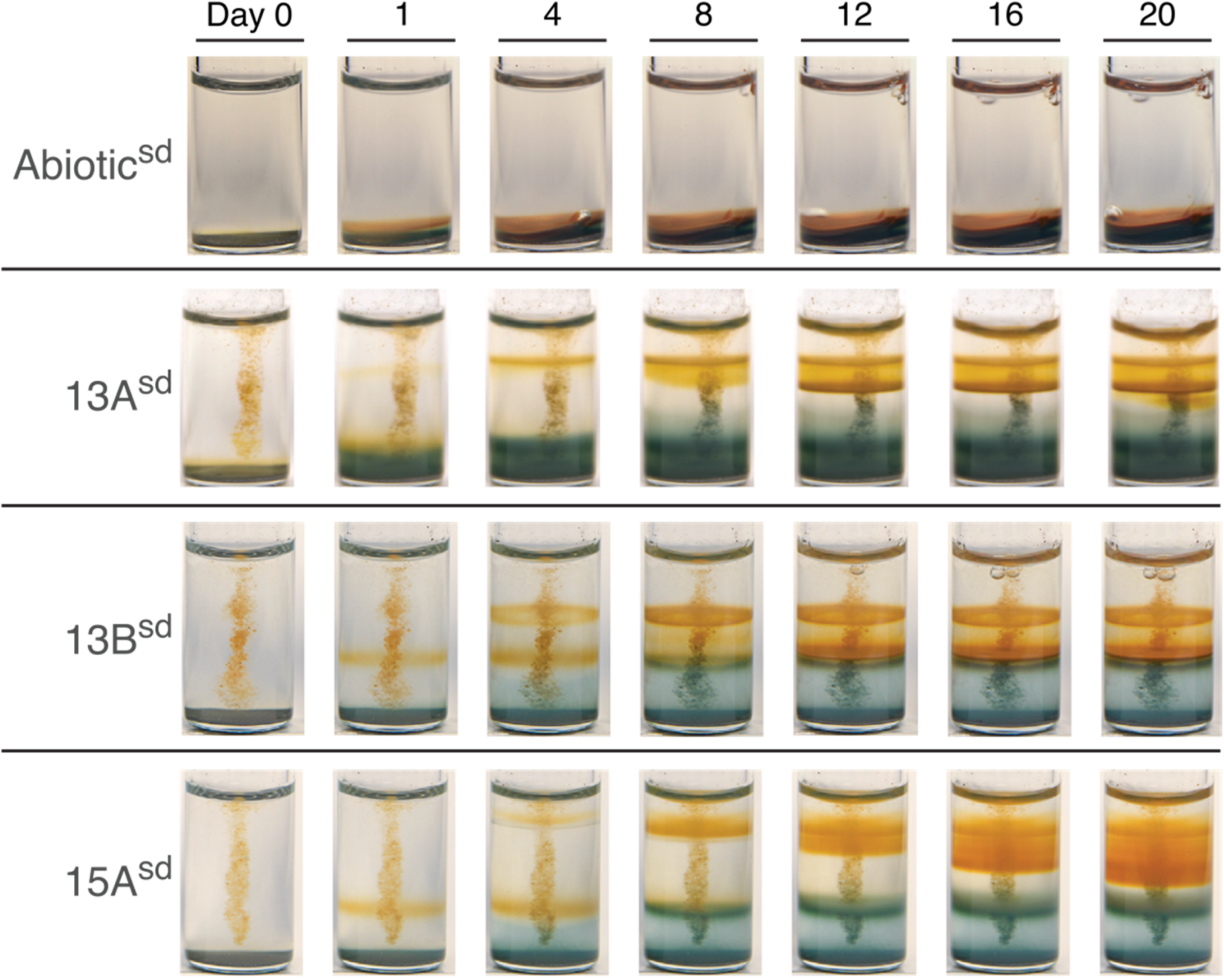
Time lapses were recorded over a 20‐day period, starting immediately after gradient tube inoculation (Day 0); images were collected every 12 h. Each row represents a different enrichment culture in sulfate‐free gradient tubes and each column is a different time point during the incubation. The vertical line of debris seen in most tubes is an artefact from inoculation, except for the abiotic control (top row), which has no inoculum.

Timelapse imaging (Figure 2) demonstrated that the cycling of the iron‐oxidising fronts and mineral precipitation in all three sulfate‐depleted lineages was highly dynamic. Monitoring the appearance of gradient tubes throughout their incubations revealed that fronts of iron oxidation appeared successively, meaning that we did not observe more than one front of iron oxidation starting at a given time, as can be most easily observed during the establishment of the first band of iron (oxyhydr)oxides in Figure 2. Additionally, all of the sulfate‐depleted lineages established a distinct band of iron oxidation within the first 24 h after inoculation. Within 8 days, cultures began to develop additional domains of growth or extended mineral formation above or below the initial growth band. In 13B^sd^ and 15A^sd^, a second band would form above the first band by Day 4. In contrast, it usually took 10–12 days for a second band to appear in 13A^sd^, and unlike the other lineages, the new band would always appear below the first band. Spatially resolved oxygen measurements along the vertical axis of gradient tubes (Figure S3) demonstrated that the region below the initial growth band is largely depleted in oxygen, suggesting that organisms growing in this region are microaerobic or anaerobic. While 13A^sd^ and 13B^sd^ always maintained 2 bands, the number of additional bands that formed in 15A fluctuated between 2 and 4 from generation to generation, and at times, the distinction between bands was difficult to assess due to the proximity and haziness of certain bands (hence the discrepancy in tube appearance between Figures 1 and 2). This pattern in 15A^sd^ resembles that of *Sideroxydans lithotrophicus* ES‐1, a freshwater microaerobic FeOB, though a more thorough investigation and isolation of the species in 15A^sd^ is necessary to establish whether or not this resemblance is merely superficial (Druschel et al., 2008).

The generally discrete nature of some of the iron (oxyhydr)oxide bands in the sulfate‐free gradient tubes is a feature consistent with the presence of FeOB (Lueder et al., 2018), or at the very least, that the localisation of precipitation was caused by biological activity (Jain et al., 2021). A biosignature of some FeOB is the presence of structured iron (oxyhydr)oxides that are undeniably biogenic in origin, taking on forms like twisted stalks and tubular sheaths that, according to current understanding, cannot be formed abiotically. That said, there are also isolated FeOB that make iron (oxyhydr)oxides that are superficially indistinguishable from those formed abiotically (Beam et al., 2018; Mori et al., 2017), and so the absence of obviously biogenic iron (oxyhydr)oxides does not necessarily translate to an absence of iron‐oxidising microorganisms.

**FIGURE 3:**
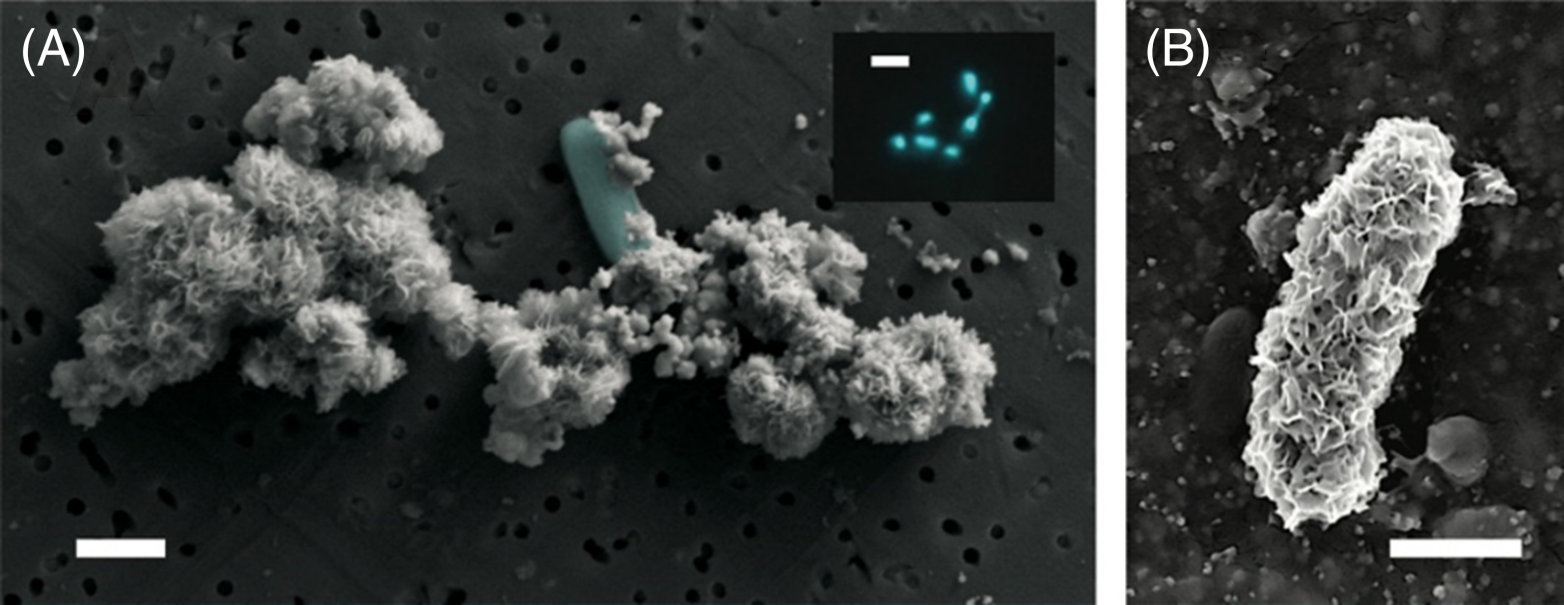
Representative images of sulfate‐depleted incubations illustrating cells (cyan) associated with (A) and encrusted with (B) iron (oxyhydr)oxides. Cultures were grown in liquid, sulfate‐free media under a headspace of 8% O_2_/10% CO_2_/baln. N_2_ for 1 week prior to preparation for imaging. Samples for SEM were prepared anoxically. Aliquots were fixed in 2.5% glutaraldehyde, collected on a 0.22 μm‐pore nucleopore filter, then gradually dehydrated in increasing ratios of ethanol to ASW until filters were in 100% ethanol. Filters were then critically point dried and sputter‐coated with a 5 nm layer of gold, followed by immediate imaging. Inset (A) is an epifluorescence image of sample from 13A, where cells (cyan) are stained with Syto13. Scale bar = 1 μm, except in inset epifluorescent micrograph, where scale bar = 2 μm.

We did not find any obviously biogenic iron (oxyhydr)oxide structures with scanning electron microscopy (Figure 3), though we did observe several instances of cells (confirmed by epifluorescent microscopy, Figure 3A, inset) showing a physical association with iron (oxyhydr)oxides (Figure 3A) or cells that appeared completely entombed by iron (oxyhydr)oxides (Figure 3B). However, in the absence of known structures and due to the lack of another mineralogical or geochemical signature for biogenic iron (oxyhydr)oxides, we cannot say for certain whether these minerals are indeed biogenic in nature.

### Sequencing

**FIGURE 4:**
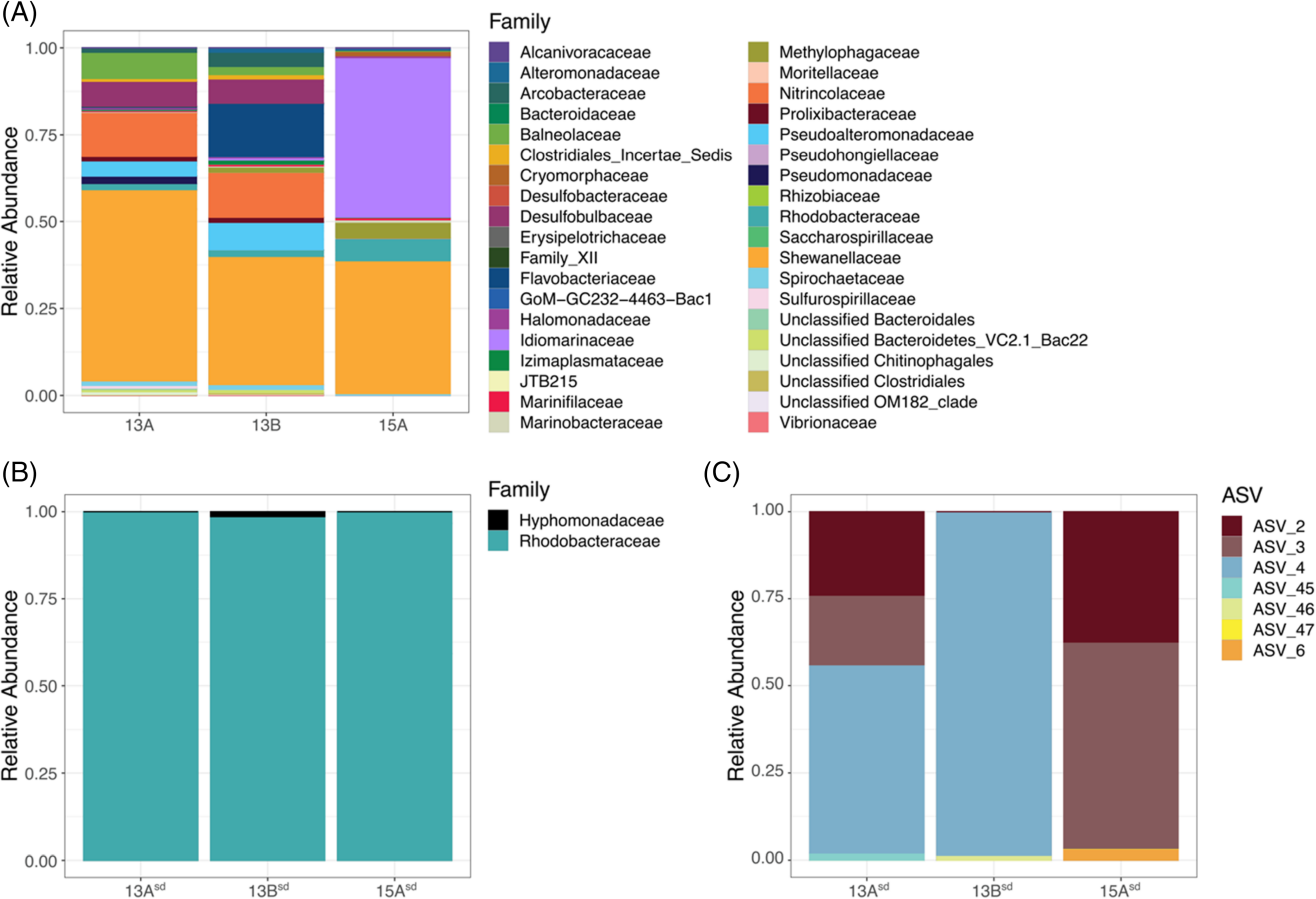
Bacterial community composition of sulfate‐containing enrichment cultures (five generations after original inoculation with environment material) and sulfate‐depleted cultures (five generations after inoculation with material from sulfate‐containing cultures). Taxonomic diversity based on partial 16S rRNA sequences is represented at the phylum level for sulfate‐containing (A) and sulfate‐depleted communities (B), and at the level of amplicon sequence variants for sequence depleted communities (C) as well. Relative abundance is represented as the fraction of total effective bacterial sequences per sample.

16S rRNA sequencing revealed that the bacterial community composition and relative abundance changed drastically after moving cultures to sulfate‐depleted media (Figure 4). Common to all three sulfate‐containing enrichment communities was a high abundance (>40% of total ASVs) of *Shewanella* spp. (Figure 4A), a genus known for its wide breadth of electron acceptors it can use, including ferric iron (Beblawy et al., 2018; Fredrickson et al., 2008). 15A was also enriched in *Idiomarinaceae* and *Methylophagaceae* (now part of *Piscirickettsieaceae*) while 13A and 13B also showed increased representation of species belonging to *Nitrinocolaceae*, *Baleonaceae* and *Desulfobulbaceae*. Some *Methylophagaceae* species are sulfide‐oxidisers, and it has been suggested that this trait may be widespread amongst members of this family (Boden, 2012; Boden et al., 2010; de Zwart et al., 1996). The possibility that the *Methylophagaceae* represented in 15A could be sulfide‐oxidising bacteria, along with the presence of known sulfate‐reducing bacteria (Kuever, 2014) (*Desulfobulbaceae*) in 13A and 13B, is consistent with the hypothesis that the iron sulfides that formed in sulfate‐containing gradient tubes (Figure S1) was the result of sulfide‐reducing bacteria reducing sulfate in the media to sulfide. All of the sulfate‐containing enrichments also had small relative abundances of *Rhodobacteraceae* species, which notably skyrocketed in abundance in the sulfate‐depleted communities (Figure 4B).

Indeed, sulfate‐depleted enrichments were markedly less diverse than their sulfate‐containing counterparts at both the taxonomic and ASV levels, with each comprised of only 3–4 ASVs (Figure 4C). These ASVs all belonged to the alphaproteobacterial family *Rhodobacteracae*, except for one ASV in 13B^sd^ that belonged to the *Hyphomonadaceae* (also part of the *Alphaproteobacteria*). The most abundant ASVs in sulfate‐depleted cultures (ASV_2 and ASV_3 in 13A^sd^ and 15A^sd^, and ASV_4 in 13A^sd^ and 13B^sd^) likely all represent strains of the same species of *Sulfitobacter* spp. (>99% sequence identity with each other) according to BLAST. The other, less abundant ASVs were not shared between enrichment communities. They include ASV_45, which has 99.48% sequence identity with *Paracoccus* sp. M1‐21‐1 (HQ425319.1)—a ‘manganese‐tolerant’ bacterium isolated from black sand from the Taklamakan Desert; ASV_46, which best‐aligned (97.91% identity) with an uncultured Hyphomonad (JQ515077.1); and ASV_6, which aligned most closely with 16S sequences from *Amaricoccus* spp.

## DISCUSSION

Overall, our findings are yet another testament to the influence of the sulfur cycle in contemporary marine sediments, particularly in methane seeps. Our sulfate‐containing incubations are a closer representation of the native geochemical conditions to which these communities are adapted and demonstrated that even in a high‐iron environment, sulfur cycling dictates the magnitude and spatial range of iron‐redox cycling, largely, but not always, through the scavenging of ferrous iron as FeS minerals. Our findings were further complicated by the high likelihood of cryptic iron and sulfur cycling (Hansel et al., 2015; Holmkvist et al., 2011; Sivan et al., 2014) taking place through the reductive dissolution of ferric iron by sulfide, the re‐reduction of iron (oxyhydr)oxides by FeRB, and the shoaling of the iron‐oxidising front to depths where FeOB likely can't compete with the abiotic oxidation of Fe^2+^ by O_2_. These results also indicate that the communities we sampled are not adapted to use iron in a manner that is competitive with its scavenging by sulfide.

### *A possible role for* Sulfitobacter *spp. and other* Rhodobacteraceae *in iron cycling*

In order to disentangle any cryptic biogeochemical process, it is critical to assess what role biology may be playing and the magnitude to which it could be contributing to the overall net observed cycling of the elements in question. We are hesitant to ascribe function from 16S identity alone, except in cases where there is substantial evidence that a certain metabolic capacity is shared amongst all or most members of that clade (e.g., the *Desulfobulbaceae* and *Shewanellaceae* mentioned above). All isolated members of the *Zetaproteobacteria*, for example, are experimentally‐validated FeOB, and all Zetaproteobacterial genomes encode the putative iron oxidase Cyc2 (Koeksoy et al., 2021; McAllister, Moore, et al., 2019; McAllister, Polson, et al., 2020). However, no Zetaproteobacterial 16S sequences were identified in our samples based on analysis with ZetaHunter (McAllister et al., 2018). There are *Rhodobacteraceae* (e.g., *Rhodovulum robiginosum, Rhodovulum iodosum, Paracoccus ferrooxidans* BDN‐1) that have been reported to oxidise iron (Kumaraswamy et al., 2006; Straub et al., 1999), but as this capacity has not been thoroughly assessed throughout this family (Bryce et al., 2018; Hedrich et al., 2011), we can only cautiously speculate on the metabolic capabilities of the sulfate‐depleted communities in our study.

While the aforementioned *Rhodovulum* species are photoferrotrophs, *Paracoccus ferrooxidans* BDN‐1 (also referred to *Paracoccus pantotrophus* KS‐1) is an anaerobic FeOB (Price et al., 2018; Kumaraswamy et al., 2006), a metabolic combination which could be especially relevant to the available niches in the gradient tubes and steep oxycline of methane seep sediments alike. Relative abundance of 16S sequences does not always equate to the relative contribution of metabolic activity and elemental cycling in a system, and ASV_45—a small player in 13A^sd^—is also a *Paracoccus* sp. Likewise, the Hyphomonadaceae (comprising only 1.4% of the 16S sequences in 13B^sd^) also have 1 isolate reported to anaerobically oxidise iron (Edwards et al., 2003). While there is a lack of literature evidence that genus members of *Sulfitobacter* species (which represent the majority of 16S sequences in these communities, by abundance) are capable of iron oxidation, it is also possible that the species being isolated in our study may represent novel iron‐oxidisers, expanding the known metabolic capacity of this genus. Additionally, there have been heterotrophic *Sulfitobacter* species from Loihi Seamount reported to oxidise Mn(II) (Templeton et al., 2005), although whether this is the result of lithotrophic Mn‐oxidation or a byproduct of some other physiological process remains to be seen. Alternatively, there may be some other unknown feature that allows *Rhodobacteraceae* species to thrive in iron‐rich environments, as their abundance has been shown to positively correlate with iron concentrations (and TOC) in marine sediments (Pohlner et al., 2019).

### Relevance to Earth's past and future

Only when we moved enrichments to sulfate‐depleted media could we visually assess their inherent capacity for Fe‐redox cycling, although this also shifted the ecological and geochemical relevance of these findings to iron‐rich, sulfate‐depleted environments. Although less common in the ocean, these environments do persist in some contemporary saline environments, particularly areas with freshwater runoff (i.e., fjords and glacial melting), and in some brackish environs too. Notably, the Bothnian Sea has been an active area of research on AOM driven by iron reduction (Egger et al., 2015; Rasigraf et al., 2019; Martins et al., 2021), and this may be a more promising site to look for seep‐dwelling FeOB. In the near future, rapid sedimentation caused by anthropogenic eutrophication is predicted to spatially separate sulfate and methane in marine sediments. This would refocus controls on methane flux from S cycling to the biological redox cycling of iron as the most viable electron sink for AOM (Egger et al., 2015).

Our findings can also be integrated in re‐imagining iron‐respiring ecophysiology in oceans past. For much of Earth's history, sulfate levels were similarly depleted, only reaching close to contemporary levels around 400 million years ago. Our results imply that in sulfate‐rich environments, sulfide scavenges most ferrous iron; translated into the geological record, this would insinuate that the precipitation of iron formations would be unlikely during periods of widespread, high sulfate levels. Indeed, marine iron formations are exceedingly uncommon in Phanerozoic marine deposits, the cause of which (in part) can be inferred as the rise of a more robust global sulfur cycle. In agreement with this idea, the brief reemergence of banded iron formations in the late Paleoproterozoic could partially reflect the accumulation and persistence of oxidised sulfur following the Great Oxidation Event. Taken together, these ideas reflect the delicate feedback between the concentration, redox state, and nature of sulfur and iron. We hope that this study and others like it serve as a steppingstone for future investigations that more directly interrogate FeOB ecophysiology in the globally widespread biospheres influenced by the modern sulfur cycle and the sulfur‐depleted environs that persisted for much of Earth's past.

### Future considerations for enriching iron‐respiring organisms from sulfidic environments

In this study, we employed gradient tubes (Emerson & Moyer, 1997; Lueder et al., 2018) commonly used for the isolation of microaerobic, neutrophilic iron‐oxidising bacteria from freshwater and marine environments. However, we made two important deviations from this established technique—we extended the usual generation time from a few days to an entire month and we dramatically lowered the standard sulfate concentration in the media. These modifications will no doubt be relevant for isolating (potentially slow‐growing) iron‐cycling organisms from other sulfur‐dominated environments. However, it is important to consider how such changes may bias the taxonomic and metabolic diversity of which organisms are enriched and isolated.

Indeed, it has been reported that accumulated abiogenic and biogenic iron minerals can cause abiotic iron oxidation to outcompete biological iron oxidation within a few days of starting a new gradient tube (Lueder et al., 2018). It is possible that we lost low‐abundance iron oxidisers in our cultures—especially in our sulfate‐containing enrichments—for this reason. Likewise, longer generation times allow for the buildup of autotroph‐derived organic carbon that can promote the growth of heterotrophs like sulfate‐reducing bacteria. We also found that conventional sulfate concentrations in our media limited biological iron cycling, and so it is quite likely that we lost rarer iron‐cycling microbes by not culturing in sulfate‐depleted media from the start. This is significant considering that there have been *Zetaproteobacteria* detected at the same site from which our enrichment cultures originated (Hydrate Ridge) (Marlow et al., 2014) and at other marine methane seep environments (Nunoura et al., 2012).

Our inability to isolate or enrich detectable *Zetaproteobacteria* or other iron‐oxidising bacteria in our study and the absence of cultured iron‐oxidising bacteria reported from methane seep environments strongly suggests that traditional gradient tube methods are inadequate to isolate them. Based on this continuing gap, we recommend that future attempts at enriching and isolating iron‐cycling and particularly iron‐oxidising bacteria from similar environments employ shorter generation times between successive passages and inoculate sulfate‐depleted media at the beginning of enrichment efforts. Sulfate‐depleted media may inadvertently select for organisms that are specially adapted to sulfate‐limitation, in which case we suggest parallel enrichment efforts where sodium molybdate is added to gradient tubes containing the conventional (28 mM) sulfate concentration. Sodium molybdate is an inhibitor of metabolic sulfate reduction, but as it has been shown to have variable efficacy for different species of sulfate‐reducing bacteria (Banker et al., 2022; Biswas et al., 2009; Nunoura et al., 2012) we suggest running sodium molybdate‐amended enrichments in parallel with sulfate‐depleted enrichments.

## AUTHOR CONTRIBUTIONS

**Isabel R. Baker:** Conceptualization (lead); data curation (lead); formal analysis (lead); investigation (lead); methodology (equal); visualization (lead); writing – original draft (lead); writing – review and editing (lead). **Peter R. Girguis:** Conceptualization (supporting); funding acquisition (lead); methodology (supporting); resources (lead); writing – review and editing (supporting).

## CONFLICT OF INTEREST STATEMENT

The authors declare no conflict of interest.

## Supporting information


**FIGURE S1.** Isolation site for 13A/B and 15A. Before (left) and after (right) disturbing the seafloor surface with the suction sampler.
**FIGURE S2.** SEM and EDS analysis of black material in sulfate‐containing gradient tubes. B is magnified image of area outlined in red in A. Scale bar throughout is equal to 10 μm.
**FIGURE S3.** Dissolved oxygen profiles in sulfate‐depleted experiment, at the end of one generation (day 30). Oxygen concentrations were collected for culture replicates grown in PreSens Oxygen SensorVials (SV‐PSt3‐20 mL‐YST), with media, ZVI, and incocula volumes scaled accordingly. SensorVials were previously calibrated according to manufacturer specifications, and spatially‐resolved measurements were made with an PreSens Fibox 3 optical cable affixed to a micromanipulator.

## Data Availability

The data that in this study are available from the corresponding author, [I.B.], upon request.
