# Supplementary material for: Sulfur cycling likely obscures dynamic biologically‐driven iron redox cycling in contemporary methane seep environments
Source: Environ Microbiol Rep. 2024 May 5;16(3):e13263. doi: 10.1111/1758-2229.13263 (PMC11070330; doi:10.1111/1758-2229.13263)
Supplement: Supplementary file 1 — FIGURE S1. Isolation site for 13A/B and 15A. Before (left) and after (right) disturbing the seafloor surface with the suction sampler. FIGURE S2. SEM and EDS analysis of black material in sulfate‐containing gradient tubes. B is magnified image of area outlined in red in A. Scale bar throughout is equal to 10 μm. FIGURE S3. Dissolved oxygen profiles in sulfate‐depleted experiment, at the end of one generation (day 30). Oxygen concentrations were collected for culture replicates grown in PreSens Oxygen SensorVials (SV‐PSt3‐20 mL‐YST), with media, ZVI, and incocula volumes scaled accordingly. SensorVials were previously calibrated according to manufacturer specifications, and spatially‐resolved measurements were made with an PreSens Fibox 3 optical cable affixed to a micromanipulator. [file EMI4-16-e13263-s001.docx]

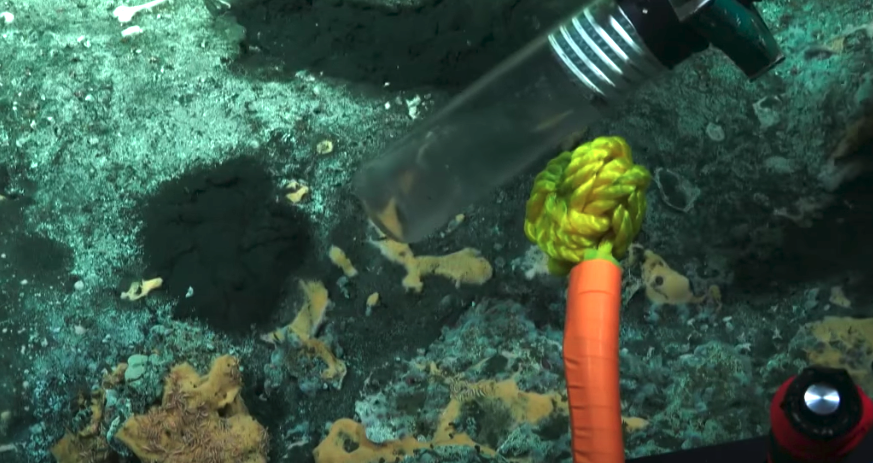

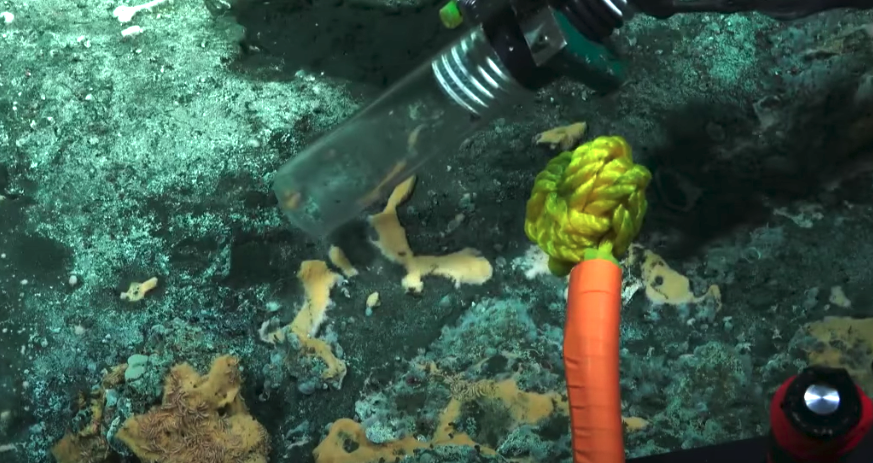


**Figure S1.** Isolation site for 13A/B and 15A. Before (left) and after (right) disturbing the seafloor surface with the suction sampler.


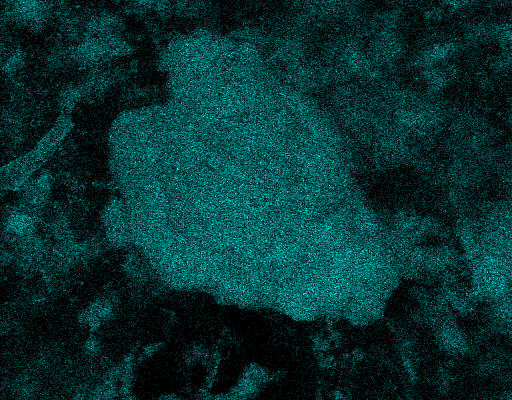

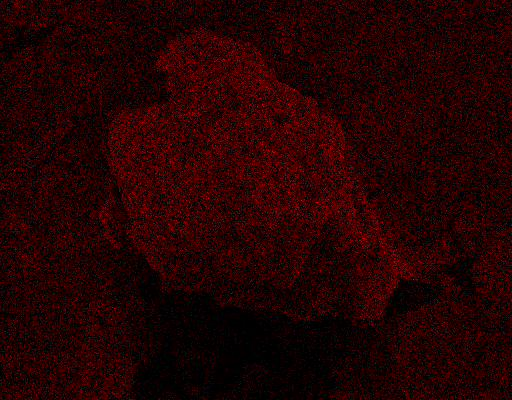


**Au**

(coating material)


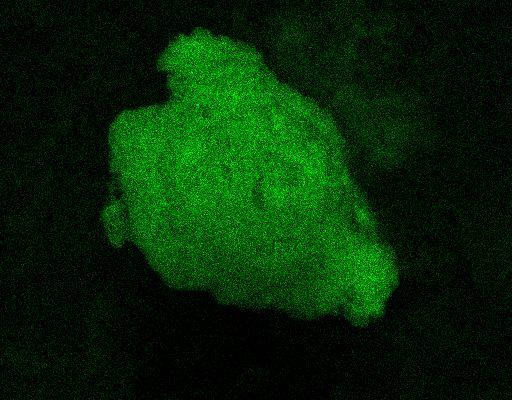

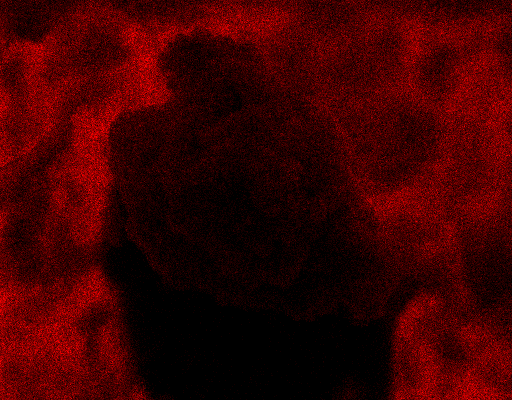

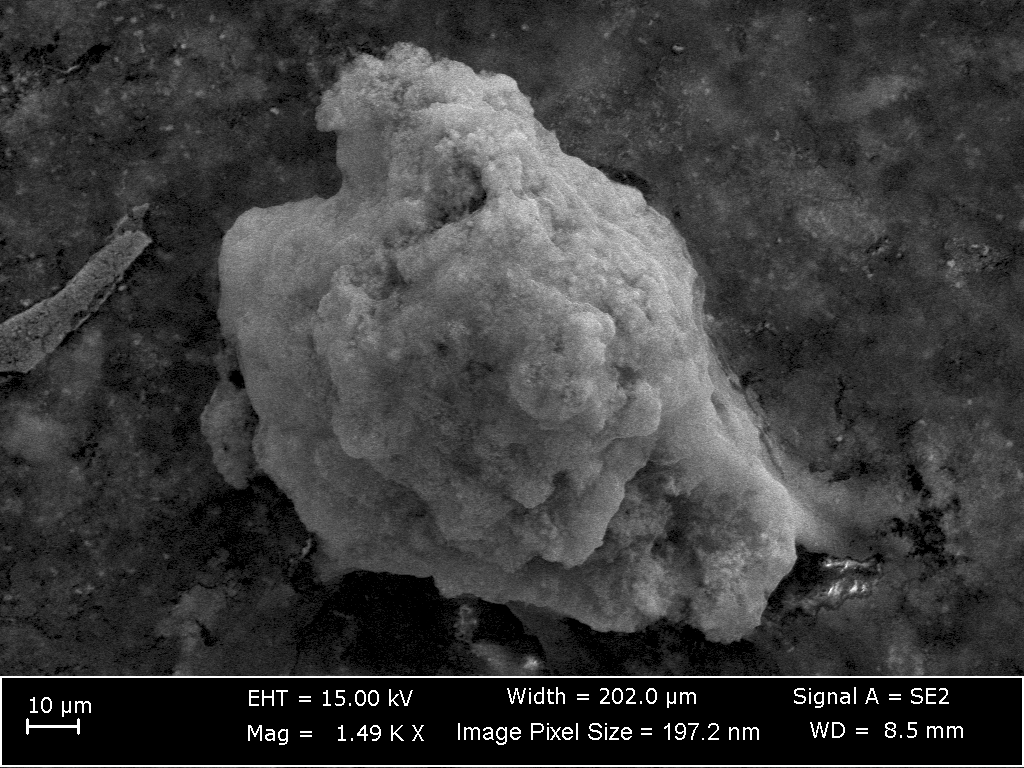

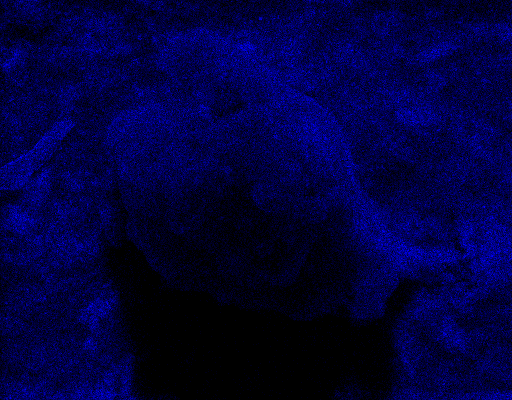


**S**

**Fe**

**C**

**O**

13A


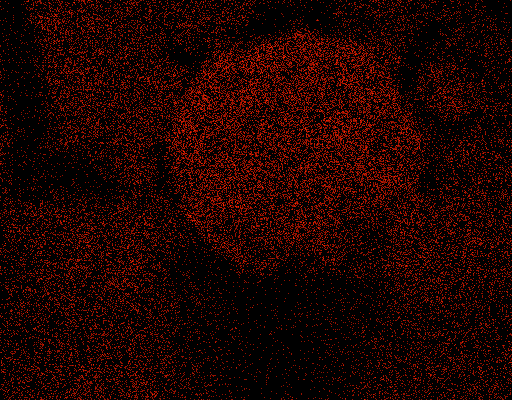

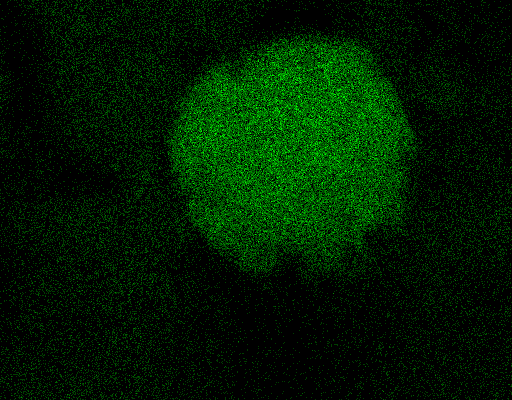

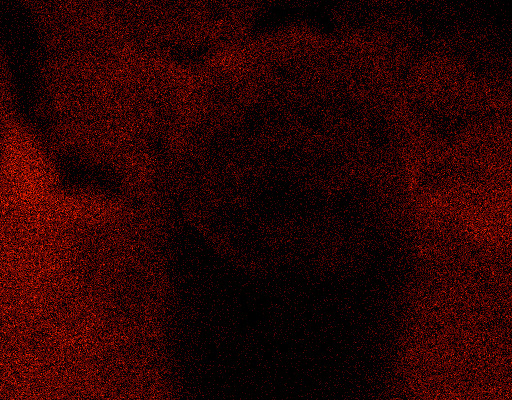

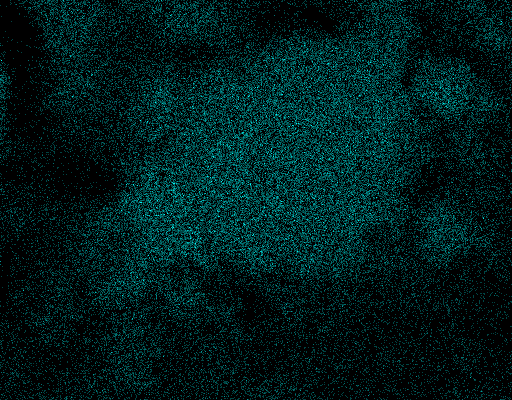

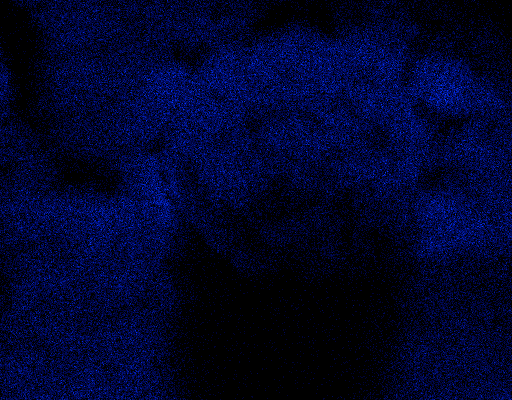

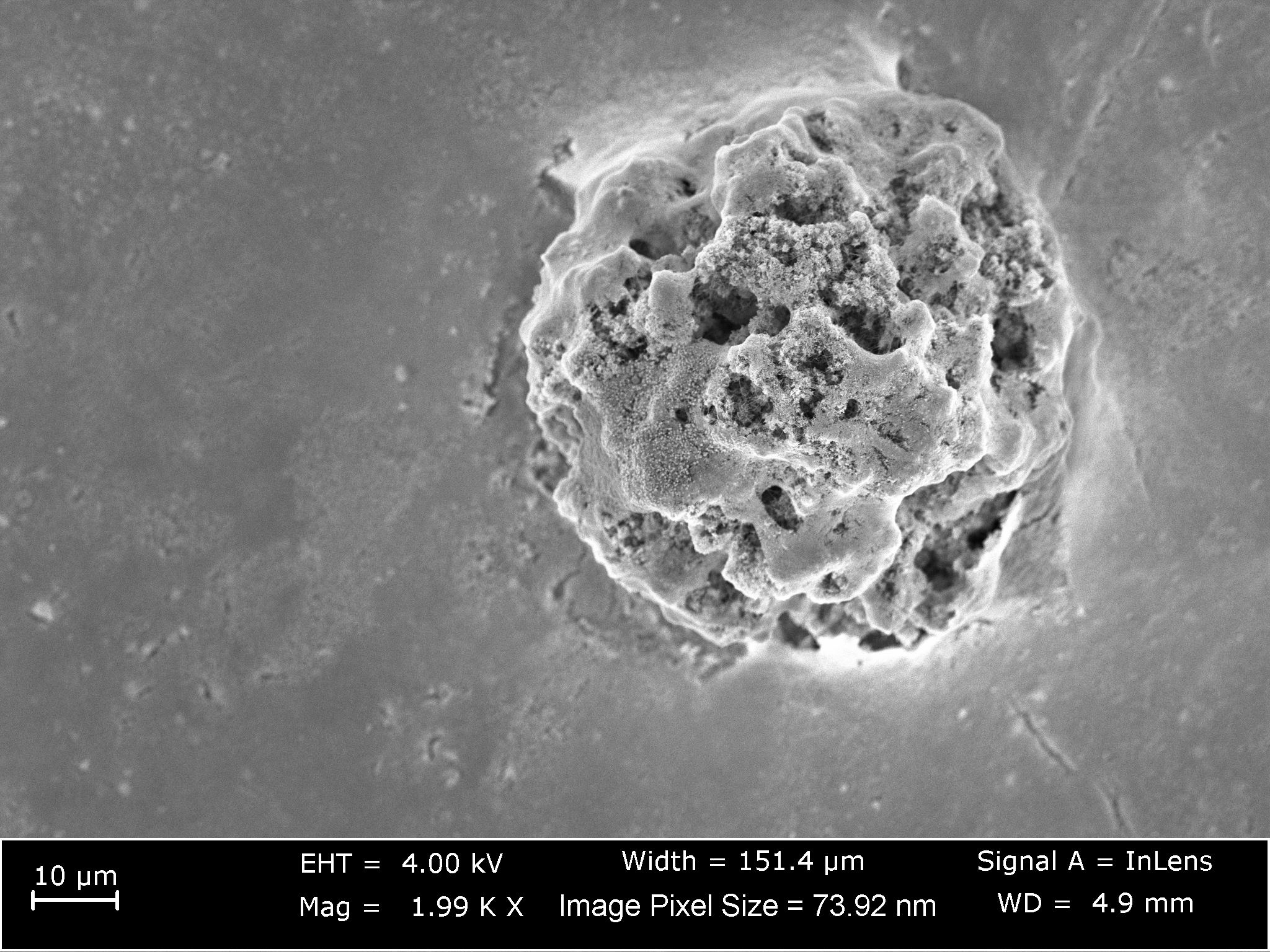


13B

*InLens*


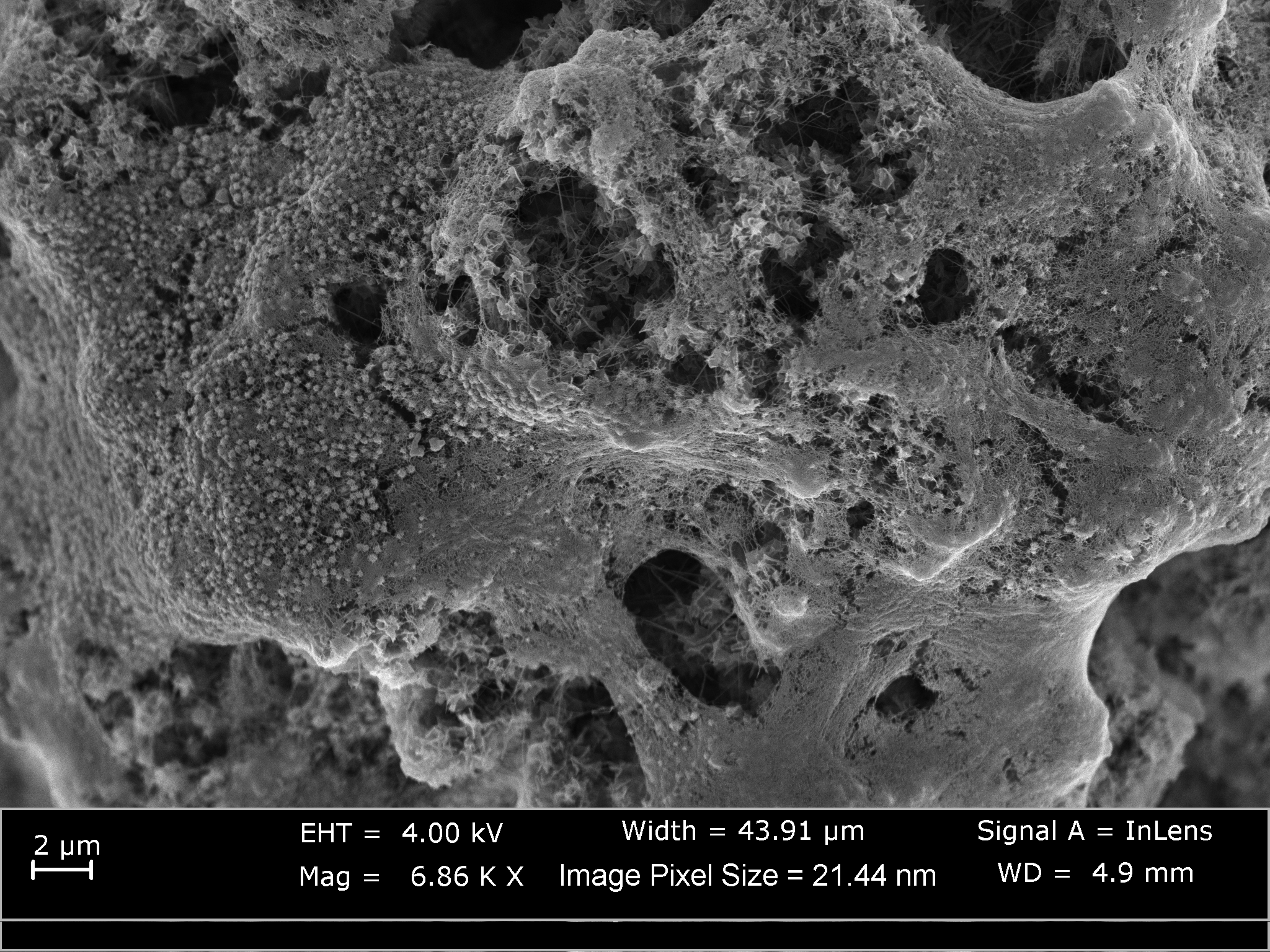


**A**

**B**

**Figure S2.** SEM and EDS analysis of black material in sulfate-containing gradient tubes. B is magnified image of area outlined in red in A. Scale bar throughout is equal to 10 μm.

**Figure S3.** Dissolved oxygen profiles in sulfate-depleted experiment, at the end of one generation (day 30). Oxygen concentrations were collected for culture replicates grown in PreSens Oxygen SensorVials (SV-PSt3-20mL-YST), with media, ZVI, and incocula volumes scaled accordingly. SensorVials were previously calibrated according to manufacturer specifications, and spatially-resolved measurements were made with an PreSens Fibox 3 optical cable affixed to a micromanipulator.
